# Editorial: Ion channels in health and disease

**DOI:** 10.3389/fphys.2022.1093210

**Published:** 2022-12-14

**Authors:** Ahmed Al-Sabi, Tarek Mohamed Abd El-Aziz, Peilin Yu, Ashlee H. Rowe, Heike Wulff

**Affiliations:** ^1^ College of Engineering and Technology, American University of the Middle East, Egaila, Kuwait; ^2^ Zoology Department, Faculty of Science, Minia University, El-Minia, Egypt; ^3^ Department of Cellular and Integrative Physiology, University of Texas Health Science Center at San Antonio, San Antonio, TX, United States; ^4^ Department of Toxicology, School of Public Health, Zhejiang University, Hangzhou, China; ^5^ Department of Biology, University of Oklahoma, Norman, OK, United States; ^6^ Department of Pharmacology, School of Medicine, University of California, Davis, Davis, CA, United States

**Keywords:** ion channels, channel modulators, potassium channels, sodium channels, calcium channels, acid-sensing ion channel 1a, channelopathies

## Membrane physiology and membrane biophysics

This Research Topic provides an update on the diverse physiological roles of ion channels and their relevance in pathophysiological processes, and as potential therapeutic targets. We selected various articles that report novel and advanced research about promising therapeutics and novel underlying mechanisms of common channelopathies.

First, Chu et al. elucidated the molecular mechanisms of intestinal glutamine uptake with a special focus on its regulation by Ca^2+^ signaling. In a pharmacological study on the small intestine of mice, they uncovered a novel Ca^2+^ regulatory mechanism of ileal glutamine transport and found that multiple Ca^2+^-permeable channels and transporters in the plasma membrane contribute to this process. The Ca^2+^ regulation of ileal Na^+^/glutamine transport expands our understanding of intestinal nutrient uptake and may be significant in gastrointestinal health and disease.

Second, Jiménez-Altayó et al. evaluated the putative vasoactive effects of two Imidazoline receptor (I_2_R) ligands, B06 and MCR5, and found that MCR5 more potently relaxes the mouse aorta of young mice than the high-affinity I_2_R selective ligands B06 and 2-BFI. Notably, the high affinity I_2_R selective ligand 2-BFI evoked marginal relaxation while agmatine, a non-selective IR ligand, did not relax the mouse aorta. Thus, they confirmed that MCR5 induced relaxation is largely independent of I_2_R activation, is only slightly modulated by endothelium-derived factors, and is mostly mediated through the activation of smooth muscle K_ATP_ and inhibition of L-type voltage-gated Ca^2+^ channels. Surprisingly, MCR5 induced relaxation is preserved in both endothelium-removed arteries and in older mice with endothelial dysfunction. Furthermore, the I_2_R ligand MCR5 is an endothelium-independent vasodilator that acts largely *via* I_2_R-independent pathways and is resistant to aging. Therefore, Jiménez-Altayó et al. propose MCR5 as a candidate drug for the management of vascular disease in elderly people.

Acidosis is a hallmark of ischemic stroke and a promising neuroprotective target for preventing neuronal injury. In another contribution, genetic manipulations showed that blockade of acid-sensing ion channel 1a (ASIC1a)-mediated acidotoxicity could dramatically reduce the volume of brain infarction and restore neurological function after cerebral ischemia (Qi et al.). However, few pharmacological candidates have been identified to show efficacy on ischemic stroke through ASIC1a inhibition. Qi et al. examined protective effects of a Psalmotoxin 1 (PcTx1) -inspired compound 5b (C5b), the highly selective and potent toxin-based ASIC1a inhibitor, in animal models of ischemic stroke *in vivo*. They found that C5b exerts significant neuroprotective effects not only in acid-induced neuronal death *in vitro* but also in ischemic brain injury *in vivo*, suggesting that ASIC1a is a druggable target for therapeutic development. Interestingly, C5b can cross the blood-brain barrier and significantly reduce brain infarct volume when administered intravenously in the ischemic animal model, highlighting its systemic availability for therapies against neurodegeneration due to acidotoxicity. These results demonstrate that C5b is a promising lead compound for neuroprotection by inhibiting ASIC1a.

Timic Stamenic et al. used a combination of patch-clamp recordings from acute brain slices, *in vivo* electroencephalogram (EEG) recordings, and wild-type (WT) and Ca_V_2.3 knock-out (KO) mice to investigate the molecular mechanisms of neurosteroid-induced hypnosis. The authors demonstrated that a recently characterized neurosteroid analog with T-channel blocking properties (3β-OH) induces hypnosis in rat pups without triggering neuronal apoptosis. In thalamic slices, the researchers found that 3β-OH inhibited spike-firing more profoundly in WT than in mutant mice. In subsequent *in vivo* experiments, intra-peritoneal injections of 3β-OH were less effective in inducing Loss of Righting Reflex (assessing the depth of hypnosis) in the mutant mice than in the WT mice, with expected gender differences. They observed sex differences in both WT and Cav2.3 KO animals, where females were more sensitive to the neurosteroid effect in comparison to males within the injection of 3β-OH. Furthermore, the reduction in total α, β, and low γ EEG power was more robust in WT than in Ca_V_2.3 KO females over time. These collective results demonstrated for the first time the importance of the Ca_V_2.3 subtype of voltage-gated calcium channels in thalamocortical excitability and the oscillations that underlie neurosteroid-induced hypnosis.

In another study Yang et al. aimed to determine whether advanced glycation of fibronectin impacts K^+^ channel activity in isolated arterial vascular smooth muscle cells. Cells were obtained from the cerebral arteries due to the potential relevance to cerebrovascular dysfunction in diabetes. In this article, fluorescence confocal microscopy was performed to measure ROS production as an indicator of acute AGE signaling acting *via* RAGE. Pharmacological experiments revealed that glycation of human plasma fibronectin (gFN) impaired both the voltage-gated K^+^ channels (KV) and large conductance Ca^2+^-activated K^+^ (BK_Ca_) channel components of total macroscopic K^+^ current. A function-blocking, anti-RAGE antibody partially reversed the inhibitory effects of gFN, suggesting the involvement of this receptor. Furthermore, gFN caused the production of reactive oxygen species (ROS) by isolated VSMCs as revealed by the fluorescent indicator, DHE. Evoked ROS production was attenuated by the RAGE-blocking antibody. Dysregulation of K^+^ channels was shown to result from disruption of physiological mechanisms, in particular the regulation of BK_Ca_ channel activity by integrin-ECM interactions, along with the pathological generation of ROS by gFN.

Finally, pathogenic variants in *KCNQ2* encoding Kv7.2 potassium channel subunits have been found in patients affected by widely diverging epileptic phenotypes, ranging from Self-Limiting Familial Neonatal Epilepsy (SLFNE) to severe Developmental and Epileptic Encephalopathy (DEE). Understanding the pathogenic molecular mechanisms of *KCNQ2* variants and their correlation with clinical phenotypes is important for the clinical management of these patients. No previous study has yet been performed on *KCNQ2* splice-site variants. Mosca et al. investigated genetic, biochemical, and functional effects of two variants that were found in SLFNE or DEE patients and both affected nucleotides at the *KCNQ2* intron 6-exon 7 boundary. Analysis of *KCNQ2* mRNA splicing in patient-derived lymphoblasts revealed that the SLFNE-causing intronic variant impeded the use of the natural splice site, resulting in a 10-aa Kv7.2 in frame deletion. By contrast, the DEE-causing exonic variant only had subtle effects on the splicing process at this site. Patch-clamp recordings in transiently transfected CHO cells and primary neurons revealed that both variants abolished Kv7.2 channel function and exerted strong dominant-negative effects when co-expressed with Kv7.2 and/or Kv7.3 subunits. The study suggests that the two variants differentially affected the splicing process at the intron 6-exon 7 boundary and led to the synthesis of Kv7.2 subunits showing a differential sensitivity to PIP_2_ and CaM regulation. More studies are needed to clarify how such different functional properties contribute to the widely-divergent clinical phenotypes.

Taken together, this article collection highlights ongoing research on the pathophysiological role and the therapeutic potential of ion channels, which still offer many unexplored opportunities for improving human health.

